# MOTANA: study protocol to investigate motor cerebral activity during a propofol sedation

**DOI:** 10.1186/s13063-019-3596-9

**Published:** 2019-08-28

**Authors:** Sébastien Rimbert, Denis Schmartz, Laurent Bougrain, Claude Meistelman, Cédric Baumann, Philippe Guerci

**Affiliations:** 1 Université de Lorraine, Inria, LORIA, Neurosys team, 615 rue du Jardin Botanique, Vandoeuvre-lès-Nancy, France; 2 CHU Brugmann, Université Libre de Bruxelles, Place A.Van Gehuchten 4, Bruxelles, 1020 Belgium; 3 Department of Anesthesiology and Critical Care Medicine, Universisty Hospital of Nancy, 9 Avenue de la Forêt de Haye, Vandoeuvre-lès-Nancy, 54500 France; 4 INSERM, U1116, Université de Lorraine, 615 rue du Jardin Botanique, Vandoeuvre-lès-Nancy, France; 5 CHRU Nancy, plateforme d’aide à la recherche clinique, UMDS, Vandoeuvre-lès-Nancy, 54500 France

**Keywords:** General anesthesia, Intraoperative awareness, Accidental awareness during general anesthesia, Brain-computer interface, Electrocencephalography, Event-related synchronization, Event-related desynchronization

## Abstract

**Background:**

Accidental Accidental awareness during general anesthesia (AAGA) occurs in 1–2% of high-risk practice patients and is a cause of severe psychological trauma, termed post-traumatic stress disorder (PTSD). However, no monitoring techniques can accurately predict or detect AAGA. Since the first reflex for a patient during AAGA is to move, a passive brain-computer interface (BCI) based on the detection of an intention of movement would be conceivable to alert the anesthetist. However, the way in which propofol (i.e., an anesthetic commonly used for the general anesthesia induction) affects motor brain activity within the electroencephalographic (EEG) signal has been poorly investigated and is not clearly understood. For this reason, a detailed study of the motor activity behavior with a step-wise increasing dose of propofol is required and would provide a proof of concept for such an innovative BCI. The main goal of this study is to highlight the occurrence of movement attempt patterns, mainly changes in oscillations called event-related desynchronization (ERD) and event-related synchronization (ERS), in the EEG signal over the motor cortex, in healthy subjects, without and under propofol sedation, during four different motor tasks.

**Methods:**

MOTANA is an interventional, prospective, exploratory, physiological, monocentric, and randomized study conducted in healthy volunteers under light anesthesia, involving EEG measurements before and after target-controlled infusion of propofol at three different effect-site concentrations (0 *μ*g.ml ^−1^, 0.5 *μ*g.ml ^−1^, and 1.0 *μ*g.ml ^−1^). In this exploratory study, 30 healthy volunteers will perform 50 trials for the four motor tasks (real movement, motor imagery, motor imagery with median nerve stimulation, and median nerve stimulation alone) in a randomized sequence. In each conditions and for each trial, we will observe changes in terms of ERD and ERS according to the three propofol concentrations. Pre- and post-injection comparisons of propofol will be performed by paired series tests.

**Discussion:**

MOTANA is an exploratory study aimed at designing an innovative BCI based on EEG-motor brain activity that would detect an attempt to move by a patient under anesthesia. This would be of interest in the prevention of AAGA.

**Trial registration:**

Agence Nationale de Sécurité du Médicament (EUDRACT 2017-004198-1), NCT03362775. Registered on 29 August 2018. https://clinicaltrials.gov/ct2/show/NCT03362775?term=03362775&rank=1

**Electronic supplementary material:**

The online version of this article (10.1186/s13063-019-3596-9) contains supplementary material, which is available to authorized users.

## Background

Every year, several hundred million surgeries are performed worldwide [1]. Among these surgical procedures, 0.1–0.2% of patients are victims of an accidental awareness during general anesthesia (AAGA), i.e., an unexpected awakening of the patient during a surgical procedure under general anesthesia [2–4]. The estimated number of AAGAs can increase up to 1% in high-risk practice patients, despite apparently appropriate anesthesia administration [4–6]. Considering the high occurrence of this phenomenon, new solutions need to be found to solve this issue.

Beyond the terrifying aspect of this experience, AAGA can cause severe psychological trauma, called post-traumatic stress disorder (PTSD) [7]. Seventy percent of AAGA events are complicated with PTSD, resulting in many negative repercussions on the victim’s life: anxiety, increased risk of suicide, insomnia, chronic fear, flashbacks, and lack of confidence in the medical staff [2, 3, 8, 9]. In addition, AAGAs may also generate legal claims that induce an additional cost for the hospital [2, 6].

Unfortunately, there are currently no satisfactory monitoring solutions sufficient to evaluate the depth of general anesthesia and detect intraoperative awakening [10, 11]. The anesthesiologist’s observation of clinical signs is not sufficient to prevent an AAGA during surgery [12]. New indexes using part of the cortical frontal electroencephalographic (EEG) signal (e.g., Bispectral Index, Entropy, Patient State Index) have failed to demonstrate their reliability and superiority [5, 13–15]. Indeed, current brain monitors available for intraoperative analysis of the cortical frontal EEG may not adequately reflect an attempt of movement from the patient under anesthesia, especially if paralytics (neuromuscular blocker agents) are used.

During an AAGA, there is evidence that the patient’s first reflex is to move to warn the surgical team [16]. However, patients may desperately attempt to move, without success [17] and perform a so-called intention to move. A motor imagery (MI) can be detected by recording the EEG signal over the motor cortex such as in the brain-computer interface (BCI) domain [18, 19]. Indeed, the mu (7–13 Hz) and beta (15–30 Hz) sensorimotor rhythms are characterized, before and during a MI, by a gradual decrease of power in mainly the alpha (mu) and beta bands, and after the end of the MI, by an increase of power in the beta band. These modulations are respectively known as event-related desynchronization (ERD) and event-related synchronization (ERS) or post-movement beta rebound [20–22].

If a BCI based on the MI of a patient seems feasible [23], the impact of propofol on the EEG activity, especially over the motor cortex, is still not yet fully understood. In 2016, Blokland et al. studied the effect of propofol on voluntary subjects who performed movements according to sound beeps while an increasing dosage of anesthetic was administered to them [24]. The authors described the impact of propofol on the EEG signal and showed how the BCI domain could contribute to the issue of AAGAs, but they emphasized that this approach is not a realistic situation, since the patient is explicitly asked to perform a movement. To address this issue, we have shown in a previous article that a frequent stimulation of the median nerve is a very promising approach [23]. Indeed, previous studies have shown that a painless stimulation of the median nerve induces an ERD during the stimulation while an ERS appears after the stimulation [25, 26]. Our recent study showed that a MI was more effectively detected using a median nerve stimulation (MNS). This result highlights promising classification results that would allow us to create a reliable device which can be used in the operating room. Therefore, we can imagine a routine system where the median nerve of the patient would be stimulated, and the analysis of ERD and ERS modulations of the motor cortex would be used to know if the patient has an intention to move. However, this study was conducted without propofol, and the previous results obtained need to be confirmed. The study we propose in this clinical protocol will provide the missing answers to these questions and provide insights into the design of such a BCI.

### Objectives

The main objective is to verify that ERD and ERS patterns can be detected in the cortical motor EEG signal under light general anesthesia conditions according to three different concentrations of propofol at the effect site (0 *μ*g.ml ^−1^, 0.5 *μ*g.ml ^−1^, and 1.0 *μ*g.ml ^−1^) during four different motor tasks (i.e., real movement, motor imagery, motor imagery and MNS, and MNS alone) in a randomized sequence. The secondary objective is to describe how the ERD and ERS generated by an MNS would be modulated according to three different concentrations of propofol. In addition, a combination of MNS and an intention to move will be studied to verify the hypotheses discussed in our previous article [23]. Finally, the forward-looking goal is a translational research project that will allow the development of a new monitoring device for the detection of intraoperative awareness.

## Methods

### Study design

Each voluntary subject recruited for the study will benefit from a pre-operative anesthetic assessment between 1 and 30 days before the experiment, performed by a trained anesthesiologist (PG). Only subjects exhibiting an American Society of Anesthesiologists (ASA) status of 1 with inclusion criteria (see below) will be eligible. Exclusion criteria are also listed below. On the day of the experiment, subjects should have fasted for 6 h for solids and 2 h for clear liquids. The experiment will be held in an approved location by the Agence Regionale de Santé (*n*^∘^2017-2500), in the Surgical Intensive Care Unit JM Picard, Department of Anesthesiology and Critical Care Medicine, University Hospital of Nancy-Brabois, France. In addition to the EEG cap, all of the volunteer subjects will be asked to rest in a semirecumbent (15^∘^) supine position and will be continuously monitored with electrocardiography (ECG), a non-invasive blood pressure measurement (NIBP), a pulse oximetry (SpO_2_) (GE Healthcare, Aulnay-sous-Bois, France), and an oxygen supplement delivered by nasal cannula (2 l.min ^−1^). A 24G peripheral catheter (BD Insyte Autogard, Becton Dickinson, France) will be inserted in the left forearm and continuously infused with a crystalloid solution (Isofundine *Ⓡ*, B. Braun, Melsungen, AG, Germany). Finally, the subject will be infused with propofol LIPURO 1% (10 mg.ml ^−1^, B. Braun, Melsungen, AG, Germany) using a target-controlled infusion pump with Schnider pharmacokinetic model (B. Braun Perfusor, B. Braun, Melsungen, AG, Germany) at the effect site. During the first session of the experiment, no infusion of propofol will be performed (0 *μ*g.ml ^−1^). Anesthesia will be induced by an experienced staff anesthesiologist in charge of the study (PG). Intravenous anesthesia will be discontinued if the voluntary subject experiences a loss of consciousness.

Three sessions will be conducted without any anesthetic medications and a step increase of propofol concentration at the effect site (brain): 0 *μ*g.ml ^−1^, 0.5 *μ*g.ml ^−1^, and 1 *μ*g.ml ^−1^.

**Fig. 1:**
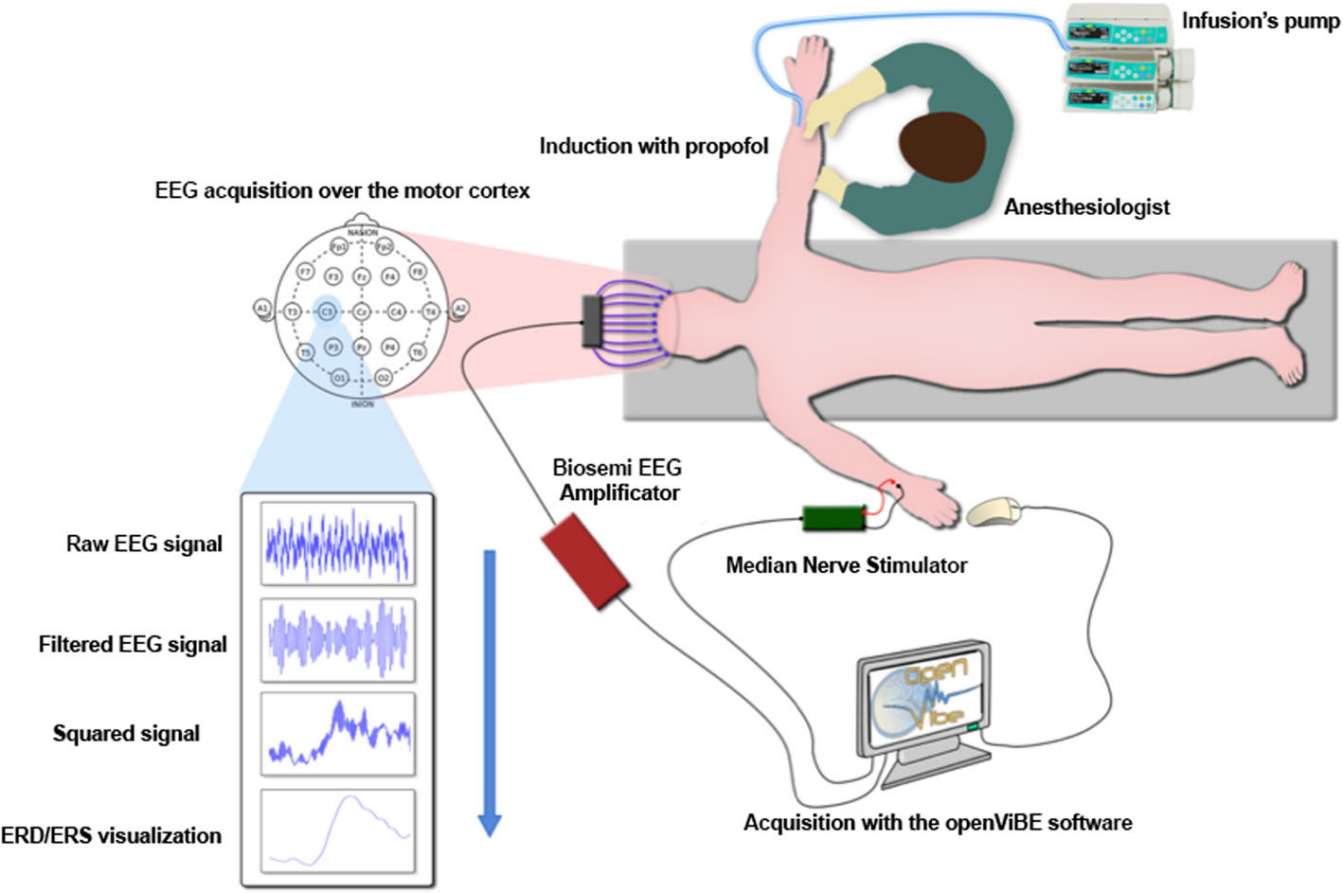
Paradigm scheme. Paradigm scheme illustrating the organization of the different sessions, tasks, and trials of the study. The study contains 3 conditions of propofol’s concentrations (0 *μ*g.ml ^−1^, 0.5 *μ*g.ml ^−1^, and 1.0 *μ*g.ml ^−1^). For each concentration, 4 motor tasks will be studied (real movement, motor imagery (MI), median nerve stimulation (MNS), and MI + MNS). All motor tasks will start with a sound beep and be followed by a resting state. 50 trials per motor task will be performed

**Fig. 2:**
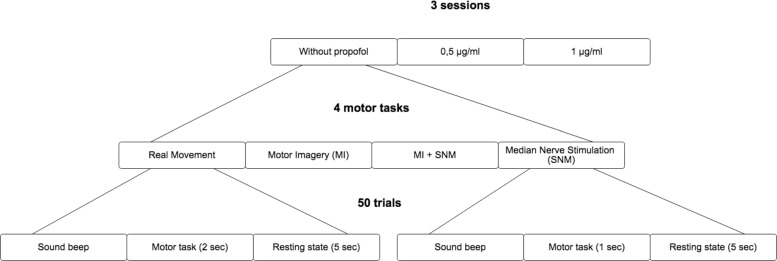
Flowchart of experimental scheme. The healthy volunteer subject will be lying down and fitted with an EEG helmet with 128 sensors, 32 of which will be placed at the level of the motor cortex. The signals will be recorded using the OpenViBE software. The infusion of propofol will be performed by an infusion pump with a concentration target

**Fig. 3:**
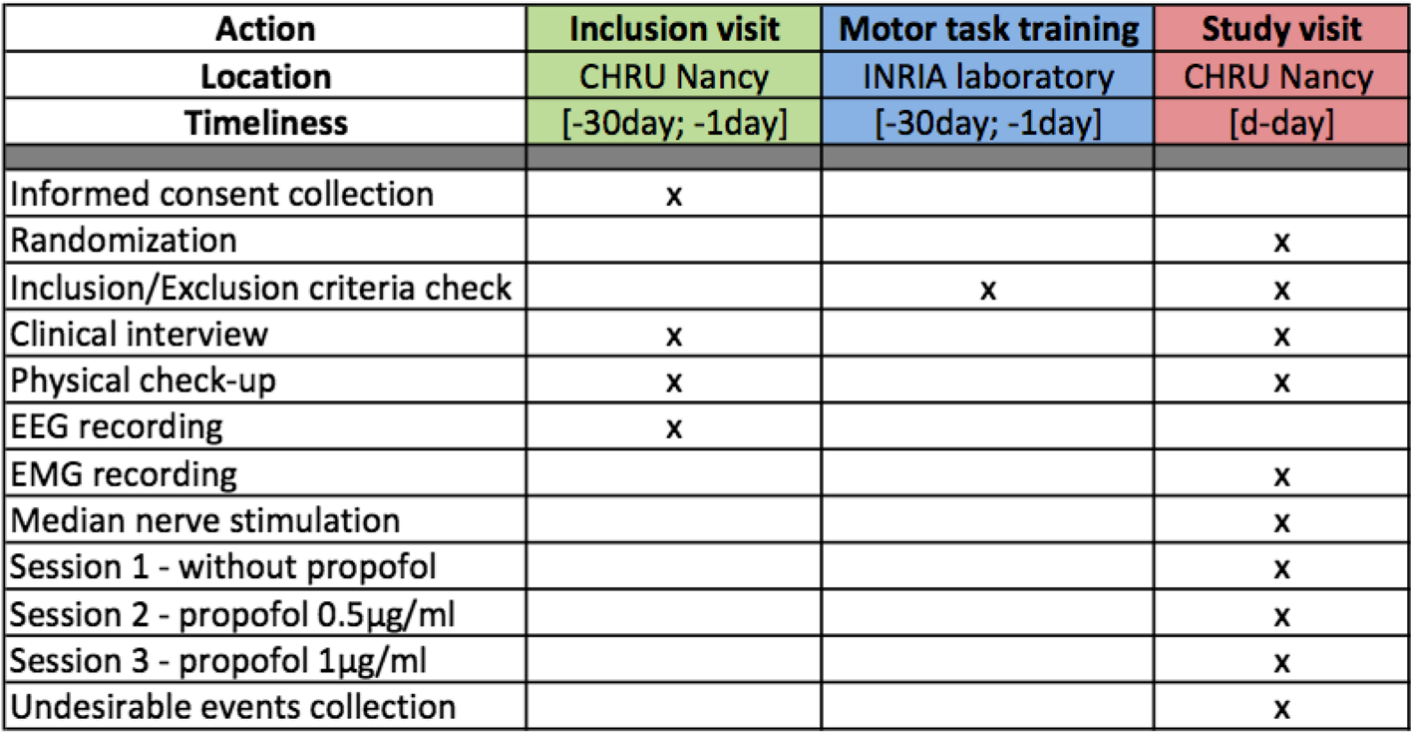
Schedule of enrollment, interventions, and assessments

For each concentration of propofol, the cortical motor EEG signal will be recorded in several sessions corresponding to the four different motor tasks: during a right-hand voluntary real movement (RM), during a right-hand kinesthetic MI, during a right-hand MNS, and during a right-hand kinesthetic MI followed by a right-hand MNS (MI + MNS). The motor tasks will be performed in a random order according to a computer-generated randomization table. Each motor task will be composed of 50 trials. The four motor tasks will be randomized for each subject in order to avoid fatigue, gel drying, or other confounding factors that might have caused possible biases in the results. At the beginning of each run, the subject will remain relaxed for 15 s. Subjects will be asked to keep their eyes closed (Fig. 1). Figure 2 is the flowchart of the experimental scheme. Figure 3 is the schedule of enrollment, interventions, and assessments. The Standard Protocol Items: Recommendations for Interventional Trials (SPIRIT) checklist is provided as Additional file 1.

Each trial will be conducted with the following steps: (1) a sound beep indicating to the patient that he/she must perform the action, (2) the action (RM or MI), and (3) a resting state period of a few seconds. During the MNS motor task, the delay will be shorter than 1 s because the stimulation will be very short (0.1 ms) and the motor patterns (ERD and ERS) will also be generated in the EEG. The right-hand median nerve will be stimulated in the same way as for measurement with a conduction velocity or for an evoked potential. The stimulation is a painless transcutaneous stimulation using the specific Micromed device Sd Ltm Stim Energy (Micromed, Mâcon, France). The stimulus intensity will range between 3 and 14 mA. The stimulation duration will be 0.1 ms with a frequency of 5 Hz. We will put the two stimulation electrodes on the right-hand wrist according to the standards [25, 28].

After all three sessions have been completed, the subject will have a 30-min rest period. Then, the subject will complete a 10-min post-experimental questionnaire. Then he/she will complete two surveys of street abilities to assess the subject’s ability to leave the hospital safely: the Chung questionnaire (Appendix 3) and the Aldrete questionnaire (Appendix 4). These two evaluation criteria will be validated by the scientific community and are commonly used for outpatient surgery. After completing the study, the subject will be contacted over the phone 24 h after the experiment by the principal investigator to ensure that everything is fine.

### Study population

In this clinical protocol, we have chosen to conduct the experiment on 30 healthy volunteers and not on patients. To ensure the completion of the study, we have designed a flyer that will be distributed on social networks. Participation in the study will also be compensated up to 80 euros. Indeed, in the case of patients, this would disrupt the functioning and organization of planned operations within the hospital. In addition, healthy volunteers will be able to be trained for the task much more easily than would be the case for patients going through surgery. Healthy volunteers will be exposed to the well-known risks of limited general anesthesia with propofol because the dosages used in this clinical protocol are lower than those that induce a loss of consciousness in healthy subjects. The doses have been determined in order to prevent the subjects from being at any risk of loss of consciousness, which will allow them to perform the required motor tasks properly. Finally, the study population will only be male, because anesthesia should be avoided for pregnant women, and the detection of pregnancy would complicate the inclusion of subjects and generate additional costs. Female volunteers are initially excluded from the study due to the extra costs generated by the need for pregnancy tests (blood tests) not budgeted. However, in case of significant results in this preliminary study, female volunteers will be subsequently included to confirm the initial findings and make the results more generalizable. Moreover, this study only concerns right-handed people, as the literature has shown significant differences between left-handed and right-handed people. The healthy volunteers will be remunerated with 80 euros in the form of a purchase voucher.

#### Inclusion criteria

To be included in the study, a person must:

- Have received full details of the research organization and signed our informed consent;
- Be aged between 18 and 28 years old;
- Be a man;
- Have a body mass index between 22 and 28;
- Be right-handed;
- Have carried out a clinical investigation adapted before the research is carried out;
- Be affiliated to a social security regime.

#### Exclusion criteria

The study will exclude a person who:

- Is allergic to any of the ingredients in propofol (in particular, soybean oil and egg);
- Has a known allergy to propofol;
- Has a history of an anaphylactic reaction during anesthesia;
- Is a female, due to the impossibility of checking pregnancy status;
- Is a pregnant or parturient woman or a breastfeeding mother;
- Is deprived of liberty by a judicial or administrative decision;
- Is undergoing psychiatric care, admitted to a health or social institution for purposes other than research, subject to a legal protection measure (guardianship, curatorship, protection of justice), or in an emergency situation;
- Is an adult who is unable to consent and who is not subject to a legal protection measure;
- Has a condition that may interfere with EEG recording (i.e., diabetes, polyneuropathy, epilepsy, depression);
- Is a toxic addict.

#### Randomization

The randomization table will be generated by the methodologist in charge of the study (CB). The investigator (SR) applies the randomization pre-established by the methodologist using pre-sealed envelopes and will note the sequence order of the tasks performed by the volunteer subject. Indeed, the subject may start with one of the four motor tasks: RM, MI, MNS, or MI+MNS. The experimentation is conducted openly, and the blinding is only for those who analyze the EEG. There is therefore no blind survey possible.

### Ethics and trial registration

The study will be conducted in accordance with the principles of the Declaration of Helsinki and the Medical Research Involving Human Subjects Act [29]. This study has been approved by a national ethical committee (Comité de Protection des Personnes Ile de France 1) under number CPPIDF1-2018-ND16. The experiment has also been approved by the Agence Nationale de Sécurité du Médicament (*N*^∘^ EUDRACT 2017-004198-15). Finally, the study protocol was registered on ClinicalTrials.gov (NCT03362775). All patients will give written informed consent before study inclusion and randomization. Patient participation is voluntary; the participant can request to stop participation in the study at any time.

### EEG data acquisition

EEG signals will be acquired using the *OpenViBE* platform [30] with a *Biosemi Active* Two 128-channel EEG system, arranged in the *Biosemi*’s ABC system covering the entire scalp at 2048 Hz. Among all recorded sites, some of the electrodes will be localized around the primary motor cortex, the motor cortex, the somatosensory cortex, and the occipital cortex, which will allow us to observe the physiological changes due to the RM, the kinesthetic MI, and the MNS [25, 26, 31, 32]. An external electromyogram (EMG) electrode will be added in order to verify that there was no movement during the MI task. All offline analyses will be performed using the EEGLAB toolbox [33] and Matlab2016a (The MathWorks Inc., Natick, MA, USA). Considering the large number of electrodes used in this study (128) and the purpose of this research (motor patterns over the motor cortex), we chose to use a common average referencing (CAR) performed using EEGLAB [34, 35]. The results will also be observed by applying a Laplacian filter and a mastoidal re-referencing [36]. Then, the EEG signals will be resampled at 128 Hz and windowed into 9-s epochs corresponding to 1 s before and 5 s after the motor task for each run.

We will compute the ERD/ERS% using the “band power method” [37]:

1 $$ ERD/ERS\%=\frac{\overline{x^{2}}-\overline{BL^{2}}}{\overline{BL^{2}}}\times{100} \enspace,  $$

where $\overline {x^{2}}$ is the average of the squared signal smoothed using a 250-ms sliding window with a 100-ms shifting step, $\overline {BL^{2}}$ is the mean of a baseline segment taken at the beginning of the corresponding trial, and ERD/ERS% is the percentage of the oscillatory power estimated for each step of the sliding window. A positive ERD/ERS% indicates a synchronization, whereas a negative ERD/ERS% indicates a desynchronization. This percentage was computed separately for all EEG channels. The EEG signal was filtered in the mu rhythm (7–13 Hz), in the beta band (15–30 Hz), and in the mu+beta band (8–30 Hz) for all subjects using a 4 ^*t*^*h*-order Butterworth band-pass filter.

ERD and ERS are difficult to observe from the raw EEG signal; an EEG signal expresses the combination of activities from many neuronal sources. We will use the averaging technique to represent the modulation of power of the mu and beta rhythms during the MI, MNS + MI, and MNS conditions since it is considered one of the most effective and accurate techniques used to extract events [20, 38].

### Statistics

For each trial (*n* = 50), ERD and ERS modulations will be computed in the 8–30 Hz frequency band for the [–2;6]s time window. This time window was chosen according to the ERD occurrence during the motor task (i.e., 2 s for RM, MI, and MI+MNS) and the time required for the ERS to return to the baseline [21]. For each trial, an ERD max and an ERS max will be selected in both their respective time windows ([0;2]s and [4;6]s). For the MNS task, in accordance with the literature [26, 27], the ERD ERS max will be respectively selected in [0.25;0.5]s and [2;4]s after stimulation. An average over the 50 ERDs max and 50 ERSs max will be performed for the motor tasks (MR, MI, MNS, MI+MNS) and each concentration (0 *μ*g.ml ^−1^, 0.5 *μ*g.ml ^−1^, and 1 *μ*g.ml ^−1^). These two values will be compared with each other for each motor task during the three concentrations with a Student’s *t* test (*p* value <0.05).

The ERD and ERS will also be visualized as event-related spectral perturbations (ERSPs). ERSPs allow one to visualize event-related changes in the average power spectrum relative to a baseline of 1.5 s taken 2 s before the auditory cue for all motor tasks and different propofol concentrations [39]. A surrogate permutation test (*p* <0.05; 2000 permutations) from the EEGLAB toolbox will be used to validate differences in terms of time-frequency of this ERSPs. In addition to this analysis, we will apply a false discovery rate (FDR) correction test in order to clarify how the FDR will be controlled for multiple comparisons. This test consists of repetitively shuffling values between conditions and recomputing the measure of interest using the shuffled data. It will be performed by drawing data samples without replacement and is considered suitable to show the difference for all motor tasks during different concentrations of propofol [40].

Finally, we will compute the performance of four different classification methods in a fourfold cross-validation scheme. The first one uses a linear discriminant analysis (LDA) classifier trained and evaluated using common spatial pattern (CSP) features generated from the first and last four CSP filters [41] (referred to as CSP+LDA). The CSP method is widely used in the field of MI-base BCI, as it provides a feature projection onto a lower dimensional space that minimizes the variance of one class while maximizing the variance of the other. The other three classifiers are Riemannian geometry-based classification methods. Riemannian geometry-based methods work with the covariance matrices of each trial, which lie on the Riemannian manifold of symmetric positive definite matrices. These features have therefore the advantage of being immune to linear transformations [42] We chose to apply a paired *t* test (two-sided) to show the significant difference in accuracy obtained for MI versus Rest and MI + MNS versus Rest with the TS + LR classifier (at *p* values <0.05).

### Study duration

For each patient, the duration of participation is 31 days, including a half-day of experimentation on the study. The experimentation on the study visit is scheduled to last approximately 3 h. The duration of the inclusion period is 24 months. The total estimated duration of the research including the time required to analyze the data is 30 months.

The Promoter has the right to interrupt the research at any time if:

- The recruitment of subjects is not appropriate;
- There are serious deviations in the protocol which have an impact on the statistical analysis of the data;
- A major problem concerning the security and rights of the subjects arises;
- The competent authority or the ethics committee so requests it.

### Data collection

Data will be collected with the use of the case report form (CRF), which will be prospectively maintained from the time the patient signs the informed consent until the completion of the study. Patient data will be anonymized; each individual will be given a unique study number (first letter of the first name and first letter of the last name, supplemented by a number assigned to the inclusion, in accordance with Reference Methodology MR001). The data monitoring committee is the Direction de la Recherche et de l’Innovation (sponsor) of the University Hospital of Nancy. An independent data monitoring committee is also nominated to assess serious adverse reactions.

The CRF will contain:

- Demographic data for each patient;
- Adverse events that may occur during the study;
- The monitoring data (blood pressure, heart rate, saturation);
- EEG data, which will be recorded electronically and stored on a computer remaining in the hospital under the supervision of the investigating anesthetist.

### Evaluation criteria

#### Principal criteria

The main evaluation outcome will be the amplitude of the ERD (event-related desynchronization)/ERS (event-related synchronization) after each motor task, within 2 s of the start signal (beep sound), before and after propofol injection and according to a pre-established increase in doses (0 *μ*g.ml ^−1^, 0.5 *μ*g.ml ^−1^, and 1 *μ*g.ml ^−1^). This amplitude will be calculated using a baseline taken before each task. The amplitudes of the ERD and ERS will be extracted. The ERDs and ERSs will be displayed from the time each power task is performed until 2 s after the task is completed.

#### Secondary criteria

The secondary evaluation criteria will include a comparison of the ERD/ERS in the three different sessions (without propofol, concentration at 0.5 *μ*g.ml ^−1^, and concentration at 1 *μ*g.ml ^−1^ at the effect site). Another secondary endpoint will be the detection of ERS after MNS. Finally, the last secondary criterion will be the statistical reliability of the detection of ERD/ERS in the primary endpoint coupled with the secondary endpoints.

## Discussion

MOTANA is an exploratory study aimed at designing an innovative BCI-based EEG-motor brain activity and would detect the intention to move of a patient during anesthesia. MOTANA is the first study to analyze the effects of a median nerve stimulation (MNS) in this context.

***Getting closer to the anesthetized state***

Since the first reflex for a patient during an AAGA is to move, a passive BCI based on the intention of movement is conceivable. Indeed, Blokland et al. have shown the feasibility of such a device [24]. However, the challenge of using such a BCI is that the intention to move from the waking patient is not initiated by a trigger that could be used to guide a classifier. In a previous study, we proposed a new solution based on MNS, which causes specific modulations in the motor cortex and can be altered by an intention of movement. We showed that MNS may provide a foundation for an innovative BCI that would allow the detection of an AAGA [23]. More particularly, we verified that MNS modulates the motor cortex by first generating an ERD during stimulation and then an ERS post-stimulation in voluntary subjects. In addition, we have discovered a new post-stimulation rebound ERS (PSR) which appears 250 ms after the stimulation in the mu and low beta bands. MNS combined with the intention to move, i.e., the MI, had a significant impact on the ERD and ERS generated by the MNS. Indeed, despite the fact that the ERD was unaltered, the PSR was almost abolished and the rebound in the beta band was diminished. Those differences have allowed a classification with highly accurate results. With these findings, we showed that a BCI based on MNS is more effective than a BCI based on a MI state versus rest [23]. Our results will be confirmed during the MOTANA clinical protocol, where the same conditions will be used on voluntary subjects sedated with propofol.

If we can find similar results in propofol-sedated subjects, we will plan to repeat the experimentation on subjects under general anesthesia with neuromusclar blockade in order to study RM intention instead of motor imagination. In a final experiment, we could combine both conditions with paralyzed and anesthetized patients in order to investigate if the combination could change the results.

***Getting closer to the implementation***

Our other perspective is to create a new way to classify our data online, with either no calibration needed or a very short one. Indeed, we require an easy-to-implement classification in order to make the hypothetical device as practical to use as possible. This also includes work on the number of electrodes required to have good results, less electrodes leading to less preparation time before a surgery. One last thing we want to study is the impact of the MNS at various times during a MI task. During this study we stimulated our subjects at the same time for the entire experimentation (750 ms after the MI task start), but in a real surgery, the MNS would intervene at different times, and the cerebral activity could be modulated differently.

## Trial status

At the time of initial manuscript submission, recruitment had started (November 2018) but has not been completed. The recruitment began on December 2018 and is expected to be completed by December 2019. The current protocol version is version 2 (September 2018) and was registered on ClinicalTrials.gov on 29 August 2018 (NCT03362775).

## Additional file

Additional file 1 SPIRIT 2013 checklist: recommended items to address in a clinical trial protocol and related documents. (DOCX 144 kb)

## Data Availability

The datasets generated and/or analyzed during the current study will be available from the corresponding author on request. Records of all patients will be kept separately in a secure place in the CHRU-Brabois hospital.

## Abbreviations

AAGA: Accidental awareness during general anesthesia
ASA: American Society of Anesthesiologists
BCI: Brain-computer interface
CRF: Case report form
CB: Cédric Baumann
EEG: Electroencephalography
ERD: Event-related desynchronization
ERS: Event-related synchronization
EMG: Electromyography
MI: Motor imagery
MNS: Median nerve stimulation
NIBP: Non-invasive blood pressure measurement
PG: Philippe Guerci
PTSD: Post-traumatric stress disorder
RM: Real movement
SR: Sébastien Rimbert
